# Analysis of *Mycobacterium africanum* in the last 17 years in Aragon identifies a specific location of IS*6110* in Lineage 6

**DOI:** 10.1038/s41598-021-89511-x

**Published:** 2021-05-14

**Authors:** Jessica Comín, María Luisa Monforte, Sofía Samper, Aragonese Working Group on Molecular Epidemiology of Tuberculosis (EPIMOLA), María José Iglesias, Daniel Ibarz, Jesús Viñuelas, Luis Torres, Juan Sahagún, María Carmen Lafoz, María Carmen Malo, Isabel Otal

**Affiliations:** 1 Instituto Aragonés de Ciencias de la Salud, Zaragoza, Spain; 2 Fundación IIS Aragón, Zaragoza, Spain; 3 Hospital Royo Villanova, Zaragoza, Spain; 4 CIBER de Enfermedades Respiratorias, Zaragoza, Spain; 5 Universidad de Zaragoza, Zaragoza, Spain; 6 Laboratorio de Investigación Molecular-UIT, Hospital Universitario Miguel Servet, Pº Isabel la Católica 1-3, planta calle, 50009 Zaragoza, Aragón Spain; 7 Hospital Universitario Miguel Servet, Zaragoza, Spain; 8 Hospital General Universitario San Jorge, Huesca, Spain; 9 Hospital Universitario Lozano Blesa, Zaragoza, Spain; 10 Salud Pública, Gobierno de Aragón, Zaragoza, Spain

**Keywords:** Biological techniques, Genetic techniques, Genomic analysis, Evolutionary genetics, Molecular evolution, Phylogenetics, Tuberculosis

## Abstract

The purpose of this study was to increase our knowledge about *Mycobacterium africanum* and report the incidence and characteristics of tuberculosis (TB) due to their lineages in Aragon, Spain, over the period 2003–2019. The study includes all the cases in our region, where all the *M. tuberculosis* complex isolates are systematically characterised. We detected 31 cases of *M. africanum* among 2598 cases of TB in the period studied. TB caused by *M. africanum* is rare (1.19%) in our population, and it affects mainly men of economically productive age coming from West African countries. Among the isolates, Lineage (L) 6 was more frequent than L5. The genotyping of these strains identified five clusters and 13 strains with a unique pattern. The isolates’ characterisation identified a copy of IS*6110* within the *moaX* gene, which turned out to be specific for L6. It will allow the differentiation of this lineage from the rest of MTBC with a simple PCR reaction. It remains to be established whether this polymorphism may limit *M. africanum* transmission. Furthermore, a mutation in the *mutT2* promoter was found as specific for L6 strains, which could be related to the high variability found for L6 compared to L5.

## Introduction

Tuberculosis (TB) is still an important cause of death, especially in developing countries^1^. All members of the *Mycobacterium tuberculosis* complex (MTBC) can cause TB, with *M. tuberculosis* being the most important. However, the strains from *M. africanum* lineages (L) are responsible for almost half of the TB cases in West Africa^2^. *M. africanum* was first described in 1968 from TB patients in Senegal^3^, showing intermediate characteristics between *M. tuberculosis* and *M. bovis*^4,5^. There are three hypotheses being considered of why *M. africanum* is almost restricted to West Africa: it is not able to compete with modern *M. tuberculosis* lineages^6^; it is adapted to the African population^7,8^; and there could be an animal reservoir, being a zoonotic disease^5,9–12^.

Regarding phylogeny, both *M. africanum* L5 and L6 have a common ancestor with the region of difference (RD) 9 deleted^13,14^. Besides, L5 split from the common phylogenetic branch before L6, and the latter also has deleted RD7, RD8, and RD10 regions. Recent studies subdivided L5 into two sub-lineages, L5.1 and L5.2, discriminated by RD711^15^.

The prevalence of *M. africanum* is rare in industrialised countries, such as in Spain; a retrospective study over 10 years (2000–2010) was published^16^. They analysed 36 cases due to *M. africanum* and concluded that most of them were immigrants from Africa, and only four cases were Spaniards. However, not all cases were searched exhaustively, considering that they did not systematically genotype the isolates.

The insertion sequence (IS) *6110*, specific to MTBC, has been used to genotype these strains since 1993^17^. Some studies analysed the location of the IS*6110* copies trying to clarify its role in the bacteria’s physiology^19,20^ or revealing that some of the IS*6110* locations are characteristic of some specific families^21,22^. However, to our knowledge, the location of IS*6110* in the genome of *M. africanum* strains has not been studied until now.

In this work, we used an epidemiological and molecular perspective to investigate the presence of *M. africanum* for the last 17 years in Aragon, Spain. The molecular analysis of these strains allowed us to identify specific polymorphisms described for the first time in these lineages.

## Results

A retrospective descriptive study of the TB cases caused by isolates identified as *M. africanum* in the Autonomous Community of Aragon was carried out. All TB cases with positive culture in Aragon between 2003 and 2019 were genotyped. Also, spoligotyping supplied the ability to distinguish among the different members of the complex, such as *M. tuberculosis, M. bovis*, and *M. africanum*. Out of 2598 MTBC cases of TB over this 17-year period, 31 cases (1.19%) have been caused by an African spoligo-family strain.

### Social and clinical characterisation of the TB cases

**Figure 1:**
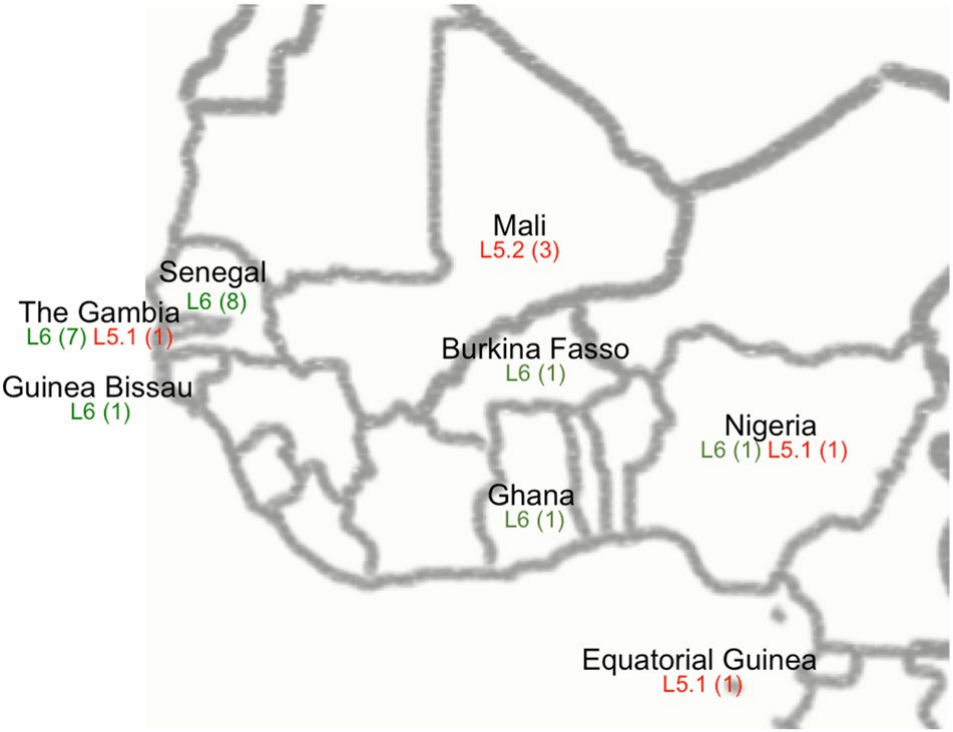
Map drawn with Adobe Photoshop CS6 (www.adobe.com) of the West African countries of origin for 26 of the cases. African countries where the patients came from are marked. The red colour indicates an L5 case of TB, and the green colour an L6 case. The number of cases is presented in brackets.

The characteristics of the cases due to *M. africanum* are detailed in Table 1. Of the 31 patients studied, 77.41% were male, the age range was between 18 and 62 years, with the largest in the 25–34-year age-group (45.16%). No cases were detected in the youngest and the eldest age groups. Regarding their origin, 27 patients were born in West African countries (87.09%), three were Spaniards (9.67%), and one patient was born in Bulgaria. The African countries of origin are detailed in Fig. 1. According to the geographical location, the number of patients who lived in an urban area was superior to those who lived in a rural area (20, 64.51% vs 11, 35.48%). At least 12 of the cases lived in our country for less than 5 years when TB was diagnosed. Attending to the location of the disease, the samples studied were: 16 sputum, four bone biopsies, six abscesses from different locations, two lymphadenopathies, and three other specimens. Near 50% of the cases (15) presented extrapulmonary disease (Table 1). For all cases, TB bacillus were susceptible to the treatment.

**Table 1:** Patients’ characteristics whose isolates were identified as *M. africanum* in Aragon from 2003 to 2019.

	N = 31	%
**Sex**		
Male	24	77.41
Female	7	22.59
**Age group**		
15–24	6	19.35
25–34	14	45.16
35–44	8	28.80
45–64	2	6.45
Unknown	1	3.22
**Origin**		
Immigrant	28^a^	90.32
Autochthonous	3	9.67
**Location of the disease**		
Pulmonar only	16	51.61
Extrapulmonary	15	48.38
**Municipality**		
Urban	20	64.51
Rural	11	35.48

^a^27 immigrants came from West African countries and one came from Bulgaria.

### Genotypic characterisation of *M. africanum* isolates

**Figure 2:**
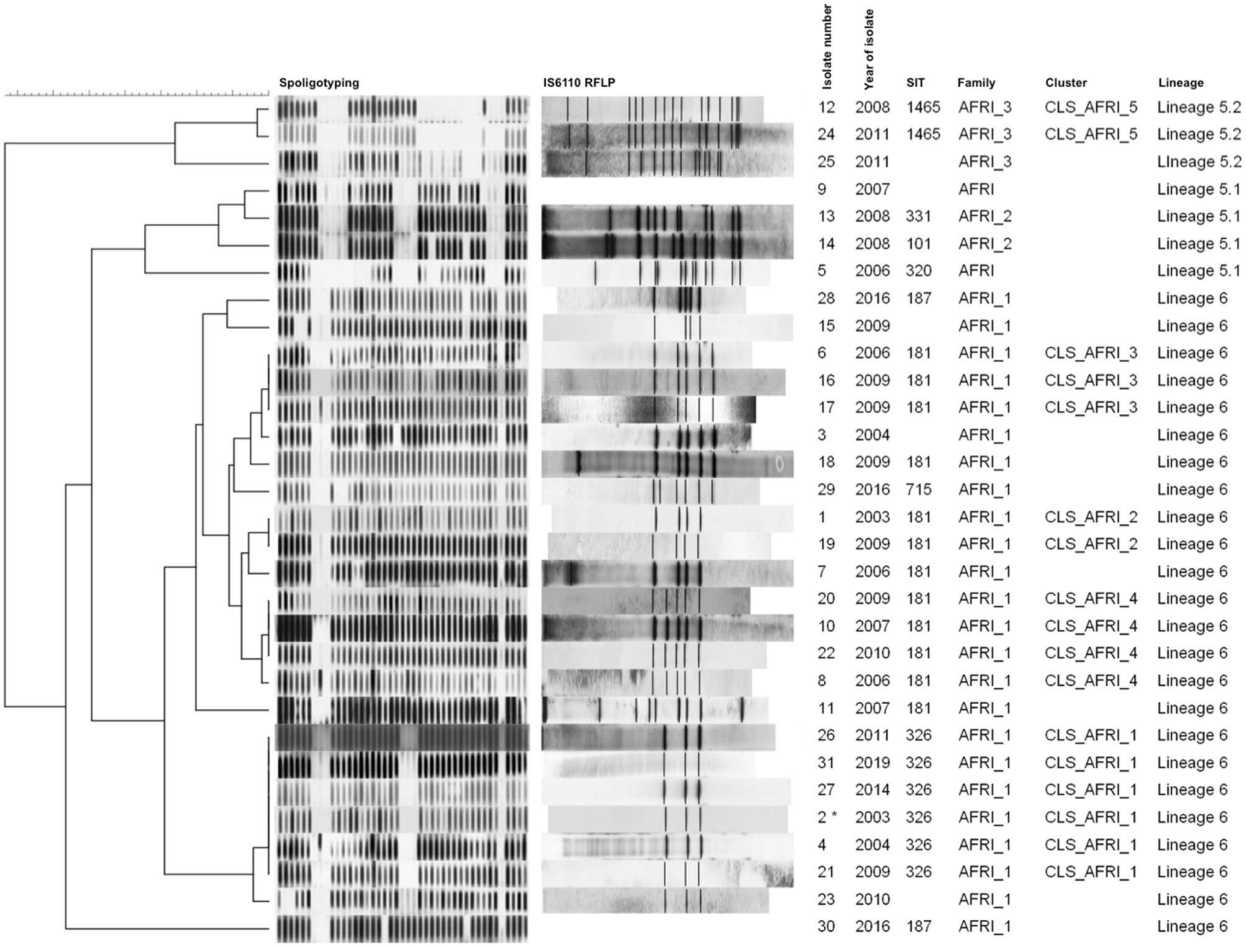
Dendrogram based on spoligotype patterns. The data shown are spoligotypes, IS*6110*-RFLP types, isolate number, year of isolation, SIT, family under SITVIT definition, and lineage of each *M. africanum* isolate in our population from 2003 to 2019. For two isolates, RFLP-type was not available, both showing a unique spoligotype belonged to AFRI and AFRI1 families. *Isolate 2 in CLS_AFRI_1 has a different location of one copy of IS*6110* despite sharing the RFLP pattern with the other five isolates included in its cluster.

The molecular analysis based on IS6110-RFLP and Spoligotyping of the *M. africanum* isolates showed five different clusters, including from two to six cases, and 13 isolates with a unique pattern (Fig. 2). Spoligotyping showed 13 different patterns, three were detected more than once (SIT 181 in 13 isolates, SIT 326 in six isolates, and SIT 1465 in two isolates) and distributed in the AFRI_1, AFRI_2, AFRI_3 or AFRI families according to the SITVIT definition^23^. To confirm the *M. africanum* lineages, the specific differential regions TbD1, RD9, RD702, and RD711 were analysed. TbD1 was present, and RD9 absent in all the isolates. Based on the study of RD702 and RD711, we could classify them into the two existent lineages of *M. africanum*. Twenty-four isolates belonged to L6 and seven to L5. There was a total concordance in the classifications obtained by spoligotyping and the RD analysis. The isolates classified as AFRI1 had the RD702 region deleted, and therefore corresponded to L6 isolates^2^. AFRI, AFRI_2, and AFRI_3 strains had RD702 present and, therefore, were considered L5. Two AFRI and two AFRI_2 spoligotype isolates had deleted RD711 and were sub-classified as L5.1. Meanwhile, the three AFRI_3 isolates had RD711 present, classifying them as L5.2^15^. The IS6110-RFLP showed a low number of IS6110 copies (≤ 6) in the L6 isolates, except one which showed eight copies. However, the six L5 isolates with available RFLP-pattern presented more than 10 IS6110 copies (Fig. 2).

Following the strain characterisation and in the context of a new assay performed in our laboratory to rapidly identify resistances and lineages, 32 isolates of our DNA collection, including different MTBC lineages, were analysed using AmpliSeq-based methodology. Two of the 32 isolates belonged to L6 (isolates 15 and 27) and three to L5.1 (isolates 5, 13, and 14). The sequence of the amplicons obtained showed five specific SNPs for L6 isolates located in *rpoB* (1163c/t^24^ and 1917a/c mutations, non-synonymous SNPs), *inhA* (233t/c, non-synonymous SNP), *katG* (609c/t, synonymous SNP), and *Rv0309* (474g/a, synonymous SNP) genes. These SNPs were reviewed and confirmed in NCBI L6 complete genomes. In the three L5 isolates studied, a SNP was present in *gyrA* (2265c/t, synonymous SNP). Finally, one specific SNP was detected in *leuB* (550t/c, non-synonymous SNP) in the isolates of both lineages L5.1 and L6 and absent in the rest of the isolates of the MTBC studied by AmpliSeq.

### Specific IS*6110* location in L6 strains

**Figure 3:**
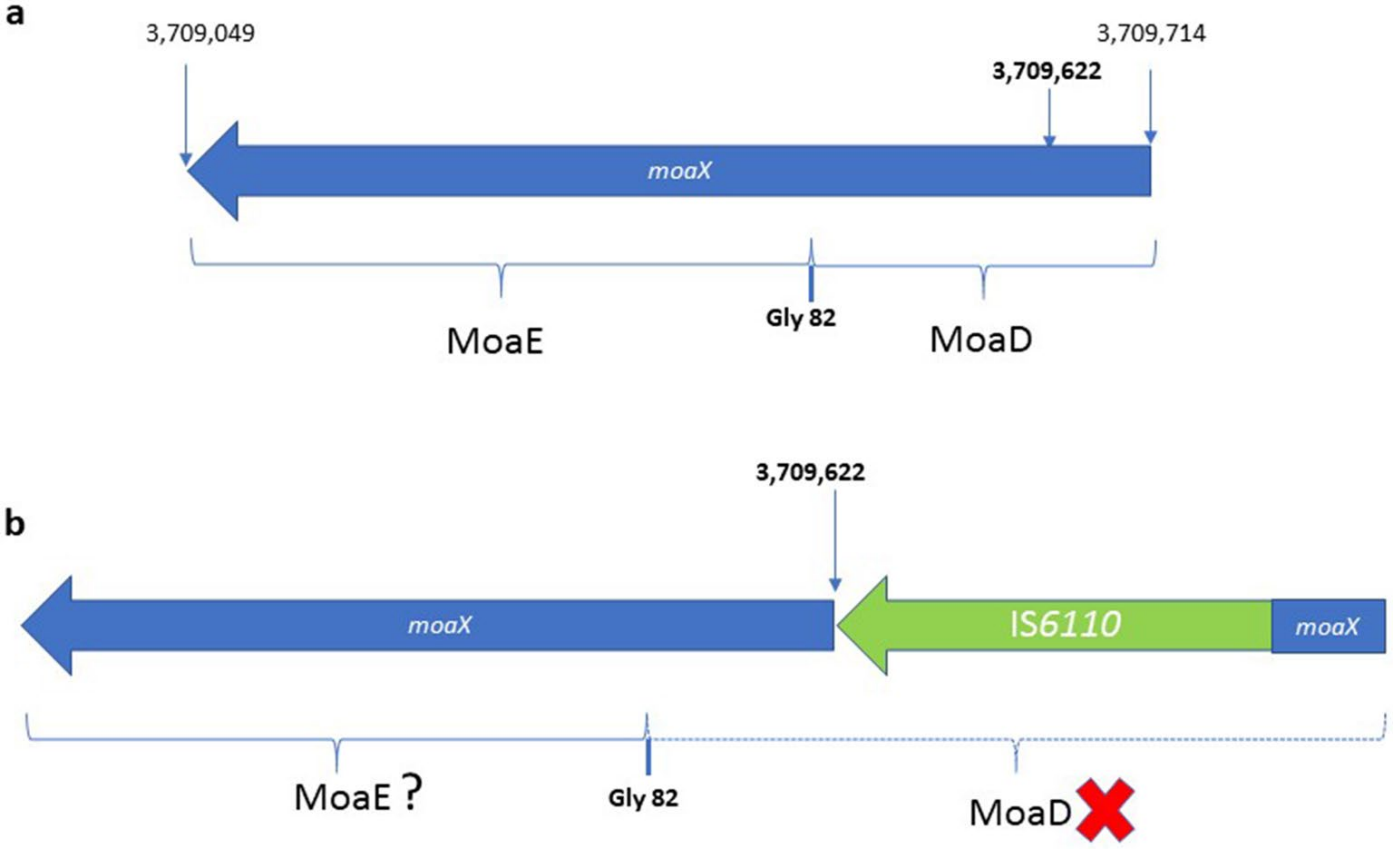
Schematic representation of *moaX* (*Rv3323c*) gene, coding for MoaX protein. The cleavage by Gly82 residue is required for the functionality of this MPT synthase. (**a**) Schema of H37Rv. (**b**) Schema of *M. africanum* L6 with IS*6110* inserted in *moaX* gene. The effect of IS*6110* is unknown for MoaE, but MoaD is going to be unfunctional. Numbers are referred to the position of the nucleotide in the genome of *M. tuberculosis* H37Rv.

**Figure 4:**
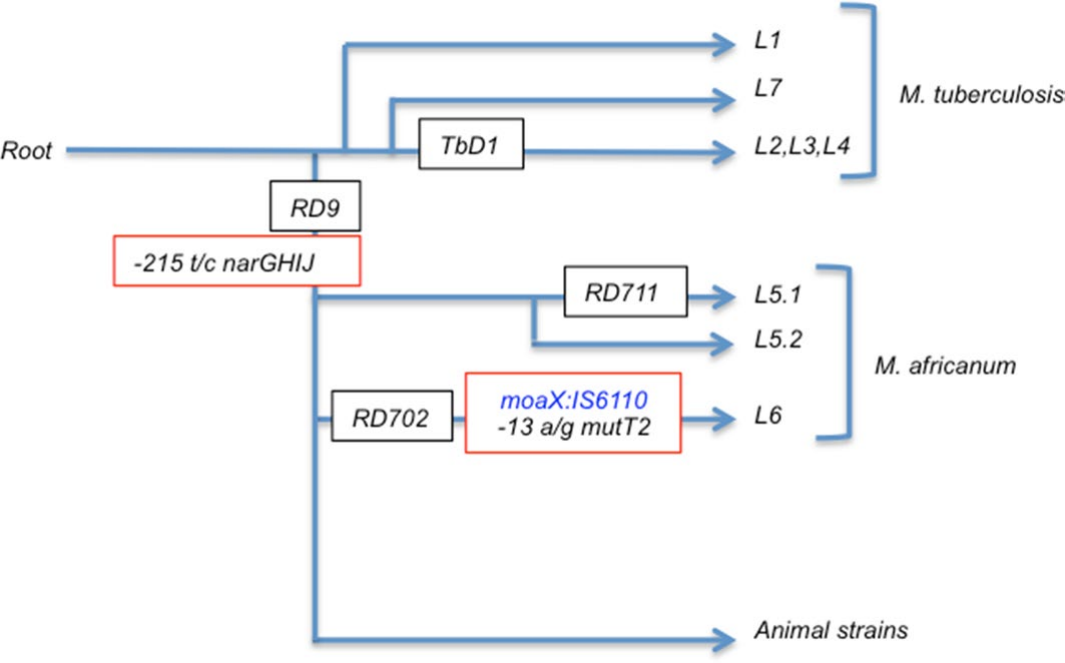
Partial evolutionary scenario of MTBC detailing *M. africanum* lineages. IS*6110* is involved in the natural evolution of MTBC, and the seemingly random transposition may have contributed to the differentiation of MTBC. Mutations and specific insertion of IS*6110* in the *moaX* gene found in this work are red-framed.

We studied the location of IS*6110* in three L6 strains (isolates 2, 15, and 21) using ligation-mediated PCR (LM-PCR) within a study of MTBC strains with a low copy number of this IS. In addition to the copy located in the DR area, three locations were detected in *Rv0963c*, *lipX:mshB* and *moaX* genes. In the three strains, one of the IS*6110* copies was located in the *moaX* gene and at identical point for all three cases (Fig. 3). Based on the results obtained, the primers moaXr (ccagtcgacgcggttgggg) and moaXd (atcgggtcattaccggcggc) were designed to verify the point of insertion of IS*6110*. The expected PCR products were 2128 bp if IS*6110* was present and 788 bp if IS*6110* was absent from the site of amplification. We sequenced the amplified fragment noting that IS*6110* was inserted at nucleotide 3709622, referred to H37Rv reference genome, flanked by three bp direct repeats (gac), as a consequence of the transposition, and located 90 nucleotides from the beginning of the *moaX* gene (*Rv3323c*) and in its same direction (Fig. 3). Further analysis showed that this IS*6110* copy was present in all our collection strains of *M. africanum* belonging to the L6 but never in our L5 strains. In addition, we observed that this location was absent in 42 isolates of low copy number studied by our group. We have also analysed this insertion point in the strains belonging to *M. africanum* L6, whose genomes are available in the NCBI (CP010334.1 and FR878060.1), verifying the presence of IS*6110* in the *moaX* gene (Fig. 4). On the other hand, to investigate the intergenic IS*6110* insertion in *lipX:mshB* as a possible specific location for *M. africanum*, we amplified the region with the primers LipX-F (gccgtttccccaatcgaatc) and LipX-R (gctcaggctctcatcgtcg). The expected fragment was 264 bp if the IS was absent and 1591 bp if it was present. The PCR results revealed the insertion in five out of nine L6 isolates tested and never in L5 isolates, which means it was not specific but frequent in L6 strains. IS*6110* was inserted at nucleotide 1300194, flanked by two bp direct repeats (tt), in all the isolates at the same point, including those in the NCBI database. However, the location of IS*6110* in *Rv0963c* was not detected in any other of the *M. africanum* isolates analysed.

### Nitrate reduction activity absent in *M. africanum* strains

Due to the location of IS*6110* interrupting the *moaX* gene, which codes for the enzymes involved in the synthesis of molybdenum cofactor (MoCo), necessary for the activity of the nitrate reductase (NR) enzyme^25^, we wanted to investigate whether this fact would be reflected in a difference in NR activity between the L5 and L6 strains. We analysed the reduction of nitrates of the *M. africanum* isolate 5 (L5) and isolate 11 (L6), and H37Rv and BCG as positive and negative controls, respectively. However, both L5 and L6 strains showed a negative result of NR activity. Both positive and negative controls were in line with expectations. Then, we analysed the sequences of *narG* and *narI* genes, which did not present any mutation to explain the L5 strain result. Nevertheless, the analysis of the *narGHIJ* promoter in four L5 isolates and two L6 isolates showed an identical mutation in − 215 (t/c), which was also present in the NCBI complete genomes of *M. africanum* (L6) and *M. bovis*. Additionally, the study of the sequenced promoter region of the *narGHIJ* operon showed a mutation in the − 13 (a/g) *mutT2* gene, upstream *narG* gene, in the two L6 isolates, which was absent in the four L5 isolates tested. This was also observed in the *M. africanum* L6 genomes included in the NCBI database.

## Discussion

This work was carried out to understand the epidemiological situation in Aragon, Spain, related to TB cases caused by *M. africanum*. This study used the data set on TB cases linked to the genotypes of the clinical isolates. The findings from this study indicate that *M. africanum* is a rare cause of TB in our region and represents 1.19% of the cases with available genotype data reported during the 2003–2019 period. A previous study regarding this causal agent^16^ reviewed the percentage detected in other countries, as Brazil, Australia and Portugal, where it represented less than 1% of the TB isolates. In their work, the authors collected information on 36 TB cases of *M. africanum* over a 10-year period in Spain. Nineteen of these isolates were from our region and therefore included in this study. The fact that they did not systematically identify the *M. africanum* isolates leads us to believe that there was an underestimation of the TB cases caused by L5 and L6 in our country. Nevertheless, our study was exhaustively conducted since 2003, identifying all *M. africanum* cases. We consider that the incidence of *M. africanum* in our country should be low given the results observed in this study, even though in higher African migration areas it could be slightly different. We hypothesised that the African lineages that are rare in our population are not adapted to transmit.

The descriptive analysis of the TB cases caused by *M. africanum* showed that most were male (77.41%) and in the 25–34 age group (45.16%). All of them were in the labour force, which could be related to being the most abundant age group among immigrants. It was more likely to occur in foreign-born people coming from West African countries (87%), being that only three cases (9.67%) were of Spanish-born people. In Esteban’s study^16^ performed in Spain, few Spaniards (7%), in contrast to immigrants, presented TB caused by *M. africanum*. The slight difference obtained by us may be because we did an exhaustive genotyping of all the cases. In our study, the patients came from different rural areas in a higher percentage (35%) than the common TB caused by *M. tuberculosis*, which occurred around 80% in urban areas^26^. These associations suggest that the epidemiology of *M. africanum* in our region is driven primarily by the migration of people from West Africa. The TB in Spaniards suggests that transmission of *M. africanum* might occur in Spain, but the possibility of TB acquisition during a trip (e.g., to West Africa) cannot be excluded, as one of the Spaniards presented a unique genotype strain. It would be of interest to continue the study in the coming years to check if the *M. africanum* strains of this work are maintained or are displaced by other MTBC strains. In previous reports, a lower transmission of *M. africanum* in comparison to L4 was observed. Nevertheless, the proportion of L5 and L6 is maintained over time, suggesting that other factors may be responsible for its continued presence in Africa^27^.

The presentation of the disease was in half of the cases restricted to pulmonary location. The extrapulmonary type of the disease (48.38%) was identified in a higher percentage than for the TB case notifications in Spain in 2017 (27.5%)^28^. Some studies showed a high proportion of extrapulmonary TB caused by L5 strains, suggesting that these strains might show a different ability to cause pulmonary disease than *M. tuberculosis *sensu stricto strains^15^. Curiously, while the extra respiratory location of the TB was high among the African cases, the three Spaniards presented only respiratory disease. The differential HLA distribution among the Mali population has been studied, and it was concluded that it might be at least partially responsible for the geographical restriction of *M. africanum* infections to West Africa^29^. The possibility that HLA could also affect the clinical presentation of the disease would explain these differences. Other studies support the hypothesis that *M. africanum* has a low degree of virulence that may be related to dissemination, rather than lung damage, during the early stages of infection^30^.

We could detect a higher percentage of L6 strains (77.41%) than L5 (22.58%), including both L5.1 and L5.2 sub-lineage cases. Other studies in Mali and Gambia also showed a higher identification of L6 among their cases^2,31^. A systematic review of current knowledge about MTBC strain diversity and geographical distribution in African regions showed a different prevalence of *M. africanum* in West African countries. It represented 8% in Nigeria, 19.75% in Ghana, 20% of the isolates in Burkina Fasso, 3.3% in Guinea, 47.10% in Guinea Bissau, and 38.4% in the Gambia^32^. Previous results published about Gambia reflected that half of their isolates were *M. africanum*, and nearly all of them belonged to L6 with SIT 181 as the most prevalent pattern. According to our results, seven out of eight *M. africanum* isolates of patients coming from Gambia were L6 isolates, and five of them had SIT 181^2^. Traore et al.^31^ determined 27.8% of the cases as *M. africanum* in Mali*,* and almost all of them (94.2% of the strains) were MAF2 (L6). However, the three cases detected from Mali in our study were L5.2.

For all cases, TB bacillus were susceptible to the treatment, although the Ampliseq method applied in seven *M. africanum* isolates detected some mutations in genes related to resistance. These polymorphisms could be specific evolutionary characteristics of the respective lineages. These results indicated that we must be cautious when reporting resistant genotypes, such as the mutations found in this study, which do not confer a resistance phenotype. Nevertheless, they could be assessed as specific for L6 (*rpoB, inhA*, and *katG* genes) and L5 (*gyrA gene*).

RFLP showed a substantial difference in the number of IS*6110* copies between the L5 and L6 strains. L6 strains carried a lower number of copies in contrast to L5 strains. Spoligotyping and IS*6110*-RFLP allowed us to detect five clusters, including 17 cases. Although each technique has low discriminatory power separately, especially among low copy number strains, it increases when considered together. On the other hand, the location of some of the IS*6110* insertion points adds differentiation capacity to the RFLP, as indicated in other publications where it has been described that RFLP analysis can underestimate the real copy number for the IS*6110* element^33,34^. In this work, the isolates 2 and 21 present three bands that seem identical when observing their RFLP pattern. However, they share two locations (DR region and *moaX*) but isolate 21 has an IS*6110* in the *Rv0963* gene, which does not share isolate 2 (Fig. 2). This indicates that it can happen in some cases that the coincidence of a band in the RFLP pattern does not imply that the IS*6110* insertion point is the same. The explanation for this would be that a small difference between the lengths of the restriction fragment generated for two different locations of IS*6110* is not appreciable in the RFLP pattern. Despite this, transmission was not considered in this study as it could overestimate the recent transmission rate.

In the context of a study of the IS*6110* location in low copy number strains, we discovered an insertion within the *moaX* gene for the L6 strains analysed, and later we verified its presence in all L6 strains but never in L5 or other MTBC families studied. A previous work studied the insertion points of IS*6110* in high-copy clinical isolates, specifically focusing on the Beijing genotype and revealed that its location in *moaX* gene was not characteristic of Beijing family^22^. Also, we found that in a previous work where the locations of IS*6110* were studied in 579 MTBC strains representatives of the major lineages circulating in Europe and Latin America, the location of IS*6110* in *moaX* was not detected in any case^21^. In all L6 strains included in our collection and the strains whose genomes are available online, the insertion point was the same. Altogether, it strongly suggests that this location is specific for L6, allowing us to differentiate this lineage from the rest of the strains of the MTBC. Within the scheme of the evolutionary stage of the tubercle bacillus, proposed by Brosch et al.^13^, we suggest the transposition of IS*6110* into the *moaX* gene when L6 is separated from the rest of the lineages (Fig. 4). Besides, the location in *lipX:mshB* was frequent in L6 strains. These results agree with previous observations, indicating that each family has preferential insertion sites^21,22,35^, which is probably related to their evolutionary relationship.

The *moaX* gene encodes a molybdopterin (MPT) synthase with *moaD* and *moaE* activity that contributes to molybdenum cofactor (MoCo) synthesis in MTBC^25^. It has been shown that there is functional interchangeability between the MPT synthase subunits of *M. tuberculosis,* and in the case of MoaX, post-translational cleavage at the Gly82 residue is required for the functionality of this enzyme^36^. According to that, the IS*6110* inserted in *moaX* gene of L6 strains is interrupting the MoaD subunit (Fig. 3). It has been described that some mutants in genes involved in molybdopterin biosynthesis had lost their ability to resist phagosome acidification^37^. In most molybdenum-containing enzymes, the metal is coordinated to the dithiolene group of MPT to form MoCo. Enzymes that utilise MoCo harness the redox properties of molybdenum to catalyse redox reactions in carbon, nitrogen, and sulfur metabolism and to reduce terminal electron acceptors for anaerobic respiration^25^. One of these enzymes is NarG, a membrane-bound respiratory NR, suggesting a potentially important role for MoCo in the metabolism of *M. tuberculosis* in vivo*.* In an anaerobic environment, many bacteria can use nitrate as a final electron acceptor. Historically, *M*. *tuberculosis* has been differentiated from *M*. *bovis* because only *M. tuberculosis* can reduce significant amounts of nitrate (NO_3_^−^) to nitrite (NO_2_^−^). NR activity occurs at a low level during the aerobic growth of *M*. *tuberculosis* and increases significantly upon entry into the microaerobic stage. When we discovered the IS*6110* insertion in *moaX* for L6 strains, we expected to find differences in NR activity between L5 and L6, but none showed NR activity. This indicates that the disruption of the MoaD subunit from MoaX in the L6 strains is not the only one responsible for the lack of activity observed in vitro. This result supports the hypothesis that homologous genes could compensate for any adaptive disadvantage of the bacteria due to the natural knockouts created by IS*6110* insertion or other mutations^25^. Looking for another explanation for this result, we analysed the operon *narGHIJ* implicated in NR activity. The first mutation described^38^ that prevented NR activity was − 215 (t/c) SNP in the promoter of *narGHIJ* operon for *M. bovis*. *M. africanum* L5 and L6 have this mutation, but also *M. canetti*, which has NR activity^39^. There is another region responsible for NR activity, the *narK2* operon. A mutation in − 10 promoter elements of the *narK2* operon reduced NR activity in BCG^40,41^. We found this mutation in the L6 strains available in NCBI but not in *M. canetti*, which had the same genotype as H37Rv. It seems that the presence of both mutations could explain the lack of NR activity we observed for *M. africanum* L5 and L6. However, in latent anaerobiosis, BCG overexpressed the *narX* gene, a fused NR^42^. Thus, a similar enzyme could play this role for *M. africanum.*

Surprisingly, the search for mutations in the *narGHIJ* promoter led us to the location of a mutation in − 13 (a/g) *mutT2* gene, upstream of this operon, in the L6 strains analysed and in the NCBI complete genomes of *M. africanum* (L6), but not in L5 strains analysed nor in other TB genomes available in NCBI. This gene was studied in the Beijing lineage as a possible cause of a major number of SNPs related to resistance^43^. It has been observed that L6 has a higher variability in its genome in comparison to L5, which could be related to a higher mutation rate^44,45^. MutT2 is involved in DNA repair, therefore the mutation detected in the *mutT2* promoter could increase the polymorphisms in L6 strains^46^.

A possible limitation of this work is that the number of strains studied was low. Nevertheless, all the isolates have been exhaustively and systematically characterised in a continuous period of 17 years. Consequently, the results objectively reflect the incidence of *M. africanum* in our region. On the other hand, genotyping methods do not discriminate enough to analyse transmission, so that whole-genome sequencing of the isolates would be more informative.

In summary, the results of this study indicate that TB caused by *M. africanum* is rare in Aragon, and the majority of the cases were in immigrants from West Africa. L6 was more prevalent, with few cases of L5. As far as we know, this is the first time that IS*6110* locations have been determined in *M. africanum* strains, which has allowed us to detect the presence of a copy of IS*6110* in the *moaX* gene in all L6 strains. Further studies on the implication of interruption of MPT synthase subunit-encoding genes in the physiology of L6 strains and its possible relationship with lower virulence would be of interest. The analysis of this location showed that it is a specific characteristic of the L6 strains, which allows us to distinguish this lineage of *M. africanum* from the rest of MTBC in a simple and fast way, using a PCR-based test.

## Material and methods

### Origin of clinical isolates

In Aragon, a north region in Spain, all MTBC isolates are genotyped for surveillance purposes routinely since 2004, but 2003 isolates are also registered in the context of a previous study. In this work, we selected all patients with a microbiological diagnosis of TB caused by *M. africanum* between 1 Jan 2003 and 31 Dec 2019. The demographic (age, sex, country of birth, years since entry to Spain) and clinical (location of disease, sputum smear status, and previous diagnosis of TB) characteristics of the patients were retrospectively reviewed.

### Genotyping

Genomic DNA was isolated using the cetyltrimethylammonium bromide (CTAB) method^47^. DNA was frozen at − 80 ºC and used in the different molecular techniques in this study. All strains were systematically genotyped by restriction fragment length polymorphism (RFLP) based on IS*6110* and Spoligotyping*.* RFLP was performed as described by van Embden et al.^17^. Spoligotyping used a commercial membrane (Mapmygenome India Limited) to hybridise with the amplicons of the direct repeats region of each isolate. The procedure was previously described^48^. The genetic patterns were analysed by Bionumerics v7.6 software (Applied Maths, Kortrijk, Belgium) and introduced into the Database of the University of Zaragoza. TB cases caused by *M. africanum* were selected retrospectively by their spoligotype, a specific intermediate pattern between those of *M. tuberculosis* and *M. bovis,* according to the SITVIT definition^23^. Isolates were considered in cluster if they carried an identical IS*6110*-RFLP pattern and the same spoligotype if they had less than five copies of IS*6110*.

### Study of differential regions

Subsequently, the presence or absence of the differential regions RD9, TbD1, RD702, and RD711 were analysed and used to classify the isolates into the different lineages of *M. africanum* L5, its sub-linages L5.1 and L5.2, and L6^15^. The PCR were performed using the following primers: TBD1fla1-F (ctacctcatcttccggtcca) and TBD1fla1-R (catagatcccggacatggtg) 2637/484 pb; RD9-flankF (gtgtaggtcagccccatcc) and RD9-flankR (gcccaacagctcgacatc) 2484/72 pb^13^; RD702-F (ccgcaacttcgagtaccttt) and RD702-R (gttgggttgctggtccat), and RD711-F (ggccgccctgctcaagaacct) and RD711-R (cctaggccggcgacgaagtg)^14^.

### Study of single polymorphisms

A panel of primers focused on genes related to resistance in MTBC, and SNPs for lineage differentiation was analysed by AmpliSeq-based methodology using next-generation sequencing**.** This panel of primers was designed to amplify the *gyrA* gene from 7302 to 9818 (2516 pb), *rpoB* from 759,807 to 763,325 (3518 pb), *rpsL* from 781,560 to 781,934 (374 pb), *inhA* promoter from 1,673,303 to 1,673,440 (137 pb), *inhA* from 1,674,102 to 1,675,011 (909 pb), *katG* from 2,153,889 to 2,156,211 (2322 pb), *pncA* 2,288,681 to 2,289,341 (660 pb), *eis* from 2,714,124 to 2,715,432 (1308 pb), and *embB* from 4,246,514 to 4,249,810 (3296 pb). In addition, other hotspots to identify the linages, specifically to identify *M. africanum* L5 (SNP in point 1377185, *Rv1234*) and L6 (SNP in point 378404, *Rv0309*), were amplified and analysed. Besides, the polymorphisms previously described for *M. bovis* in the *narGHIJ* operon were analysed by amplification of the different regions. Primers used were the following: mutT2F-2 (tccggatgatgatttacctcc) and mutT2R-2 (tccgccgggtcggggac)^43^; narG-Fw (gcccagctttgacaccatcg) and NarG-Rv (gcccagatgacgtttcgccag); NarI-Fw (tggctaccactcggaatgac) and NarI-Rv (acgatgtagggccggaacag). The detailed points are referred to as NC_000962_3.

### Location of IS*6110* insertion sites

To study IS*6110* insertion sites, a ligation mediated PCR was used as described by Prod’hom et al.^49^ to amplify one or both ends of each copy of IS*6110* and its flanking sequence. Briefly, genomic DNA was digested with *Sal*I enzyme and ligated to a linker containing a *Sal*I restriction site. The resulting template was then digested by *Sal*I. PCR was performed using ISA1 or ISA3, specific primers for IS*6110* and directed outwards from this element^50^, and the linker primer Salgd. The template was initially denatured by incubation at 95 °C for 9 min and amplified by 35 cycles of PCR (95 °C for 30 s, 70 °C, and 72 °C for 90 s) followed by a final extension at 72 °C for 10 min. Amplified products were separated by standard horizontal gel electrophoresis in a 1.5% agarose gel in tris–borate-EDTA buffer (90 mM tris, 90 mM boric acid, 2 mM EDTA) and stained with ethidium bromide. PCR products were purified, using GFX PCR DNA and Gel Band Purification Kit (Amersham Pharmacia Biotech) followed by ExoSAP-IT PCR Product Cleanup Reagent (Affymetrix), sequenced and analysed for homology with Tuberculist (http://genolist.pasteur.fr/TubercuList).

### Enzymatic assay of NR

The NR activity test was performed with actively growing cultures, which were inoculated directly into phosphate buffer supplemented with nitrate and incubated for 2 h at 37 °C. The mycobacteria were cultured on 7H10 agar supplemented with 0.2% glycerol and 10% albumin/dextrose/catalase (ADC). One L5 strain and one L6 were inoculated into phosphate buffer supplemented with 10 mM nitrate. Following 2 h of incubation at 37 °C, naphthylamide and sulfanilic acid reagents were added, and the colour was then observed^51^.

### Computer analysis

The sequences generated were aligned and compared with the sequences of *M. tuberculosis* H37Rv (http://genolist.pasteur.fr/TubercuList) and *M. africanum* complete genomes, NC_015758.1 and CP010334.1 (http://blast.ncbi.nlm.nih.gov), using the Basic Local Alignment Search Tool (BLAST).

### Ethics declarations

The permission to take informed consent was formally waived by the Comité de Ética de la Investigación de la Comunidad Autónoma de Aragón (CEICA), Spain, CI.PI18/068. No human tissues were used in the study. Once received the bacterial isolate, it was coded (NSTRAIN). The epidemiological data of the cases were sent by fax and were anonymised keeping only the code given to track the analysis of the clinical characteristics, to follow the Helsinki ethical principles for medical research involving human data. The experiment protocol followed was revised and approved by the CEICA and is in line with the Declaration of Helsinki, as revised in 2013.

## Data Availability

Sequences data reported in the present study were deposited in GenBank under accession numbers MW987573MW987574 and MW987575.
