# Author Correction: Analysis of *Mycobacterium africanum* in the last 17 years in Aragon identifies a specific location of IS*6110* in Lineage 6

**DOI:** 10.1038/s41598-025-90690-0

**Published:** 2025-03-04

**Authors:** Jessica Comín, María Luisa Monforte, Sofía Samper, Aragonese Working Group on Molecular Epidemiology of Tuberculosis (EPIMOLA), María José Iglesias, Daniel Ibarz, Jesús Viñuelas, Luis Torres, Juan Sahagún, María Carmen Lafoz, María Carmen Malo, Isabel Otal

**Affiliations:** 1 Instituto Aragonés de Ciencias de la Salud, Zaragoza, Spain; 2 Fundación IIS Aragón, Zaragoza, Spain; 3 Hospital Royo Villanova, Zaragoza, Spain; 4 CIBER de Enfermedades Respiratorias, Zaragoza, Spain; 5 Universidad de Zaragoza, Zaragoza, Spain; 6 Laboratorio de Investigación Molecular-UIT, Hospital Universitario Miguel Servet, Pº Isabel la Católica 1-3, planta calle, 50009 Zaragoza, Aragón Spain; 7 Hospital Universitario Miguel Servet, Zaragoza, Spain; 8 Hospital General Universitario San Jorge, Huesca, Spain; 9 Hospital Universitario Lozano Blesa, Zaragoza, Spain; 10 Salud Pública, Gobierno de Aragón, Zaragoza, Spain

Correction to: *Scientific Reports* 10.1038/s41598-021-89511-x, published online 14 May 2021

The Funding section in the original version of this Article was incomplete.

This work was supported by the Carlos III Health Institute in the context of two consecutive Grants FIS18/0336 and J.C. was awarded a scholarship by the Government of Aragon/European Social Fund, “Building Europe from Aragon”.

Now reads:

This work was supported by the Carlos III Health Institute (ISCIII) in the context of Grant FIS18/0336, co-financed by European Regional Development Funds of the European Commission: “A way of making Europe”. J.C. was awarded a scholarship by the Government of Aragon/European Social Fund, “Building Europe from Aragon”.

The original Article has been corrected.

